# Enhanced recovery after surgery (ERAS) protocols for total joint replacement surgery

**DOI:** 10.1051/sicotj/2023030

**Published:** 2023-10-11

**Authors:** Maria Riga, Pavlos Altsitzioglou, Theodosis Saranteas, Andreas F. Mavrogenis

**Affiliations:** 1 Second Department of Anesthesiology, National and Kapodistrian University of Athens, School of Medicine, ATTIKON University Hospital Rimini 1 12462 Athens Greece; 2 First Department of Orthopaedics, National and Kapodistrian University of Athens, School of Medicine, ATTIKON University Hospital Rimini 1 12462 Athens Greece

**Keywords:** ERAS protocols, Orthopaedics, Total joint replacement

## Abstract

The enhanced recovery after surgery (ERAS) protocols are a comprehensive therapeutic approach that prioritizes the well-being of patients. It encompasses several aspects such as providing sufficient nutritional support, effectively managing pain, ensuring appropriate fluid management and hydration, and promoting early mobilization after surgery. The advent of ERAS theory has led to a shift in focus within modern ERAS protocols. At present, ERAS protocols emphasize perioperative therapeutic strategies employed by surgeons and anesthesiologists, as well as place increased importance on preoperative patient education, interdisciplinary collaboration, and the enhancement of patient satisfaction and clinical outcomes. This editorial highlights the application of ERAS protocols in the current context of total joint replacement surgery.

## Introduction

The enhanced recovery after surgery (ERAS) protocols are a comprehensive therapeutic approach that prioritizes the well-being of patients. It encompasses several aspects such as providing sufficient nutritional support, effectively managing pain, ensuring appropriate fluid management and hydration, and promoting early mobilization after surgery [1]. The initial description of ERAS protocols was provided by Kehlet with the primary objective of expediting the process of postoperative recovery [1]. At present, ERAS protocols encompass a comprehensive range of perioperative therapies aimed at facilitating the healing process following surgical procedures. The primary objective of this intervention is to enhance the overall health outcomes of patients, while also mitigating medical expenses through a reduction in hospital length of stay (LOS). Additionally, the intervention aims to decrease postoperative death rates [1].

The advent of ERAS theory has led to a shift in focus within modern ERAS protocols. These protocols now emphasize perioperative therapeutic strategies employed by surgeons and anesthesiologists, as well as place increased importance on preoperative patient education, interdisciplinary collaboration, and the enhancement of patient satisfaction and clinical outcomes. This editorial highlights the application of ERAS protocols in the current context of total joint replacement (TJR) surgery.

## ERAS protocols in TJR surgery

The main aim of ERAS is to provide a uniform method for perioperative treatment, to improve clinical outcomes. In recent years, this method has garnered significant popularity, notably within the domain of orthopaedic surgery. The implementation of ERAS protocols in TJR enables a more efficient and improved recovery process. In previous studies [2, 3], it has been argued that the adoption of ERAS protocols can lead to a significant reduction in transfusion and mortality rates, incidence of complications and LOS among patients after TJR. It is worth mentioning that these enhancements have been reported to have no discernible effect on readmission rates within 30 days [4]. To date, it has been observed that the ERAS standards display variation among different specialties. However, there is a clear agreement that the ERAS protocols can be categorized into three distinct components: preoperative, intraoperative, and postoperative [4]. In this setting, it is crucial to undertake a comprehensive examination of the available data and regularly review proposed elements by the recommended components [5].

## Preadmission phases

### Information, education, and expectation counseling

The dissemination of preoperative information enables patients to obtain relevant knowledge and support in promptly. It can be argued that the guidelines recommend comprehensive preoperative training and instruction for patients [4]. Research has demonstrated that preoperative education for patients not only reduces preoperative anxiety but also offers advantages in terms of pain management, functional improvement, and the mitigation of all adverse events [6]. The integration of preoperative patient education and counseling has been extensively employed within ERAS protocols. Given the scarcity of available data and the presence of persuasive evidence, it is advisable, then, to undertake preoperative patient education about perioperative exercise, rehabilitation, and anticipated length of hospital stay. The ultimate objective is to reduce patient’s anxiety levels and facilitate a seamless transition throughout the discharge process from the hospital.

## Preoperative phases

### Optimization

As per the current guidelines, it is imperative to adhere to certain measures for optimal outcomes. These measures include abstaining from smoking for a minimum duration of 4 weeks, discontinuing alcohol consumption, implementing procedures for early removal of urinary catheters and addressing preoperative anemia [4]. The prevalence of preoperative risk factors, including smoking, alcohol intake, anemia, and low physical activity, remains high within the domain of orthopedic surgery. The identified risk factors possess the capacity to give rise to a range of consequences, encompassing but not limited to suboptimal wound healing, myocardial infarction, cardiac arrest, pneumonia, urinary tract infection, sepsis, acute renal failure and mortality [7–9]. Although the existing evidence is limited, preoperative optimization has promised to reduce postoperative complications and expedite patients’ discharge from the hospital.

### Fasting

It is recommended that patients consume only clear fluids up to 2 h before the administration of anesthesia. In addition, it is advised to refrain from consuming solid food for 6 h prior to the procedure [4]. Preoperative fasting is implemented as a precautionary strategy to mitigate the potential hazard of aspiration that may arise following the administration of anesthesia. Nevertheless, prolonged periods of fasting may induce catabolism and provoke a physiological reaction to surgical stress. As a result, these physiological responses may lead to the onset of insulin resistance, elevated blood glucose levels and the breakdown of muscle tissue [10]. Research has indicated that shortening the fasting period can yield several beneficial outcomes for patients, such as enhanced postoperative well-being, ameliorated insulin resistance and diminished stress reactions. The fasting strategy for numerous surgical operations has been updated according to revised recommendations stated in a study [11]. Although there is limited available data on fasting in TJR, it is crucial to pursue established standards and tailor preoperative fasting protocols based on the specific timing of the surgical procedure.

### Oral analgesia

The administration of oral analgesics during the perioperative phase is of paramount importance in the ERAS protocols. The guidelines propose the routine administration of paracetamol, non-steroidal anti-inflammatory drugs (NSAIDs) and oxycodone. ERAS protocols place a high priority on minimizing the use of opioids after surgery in order to minimize the potential negative consequences. However, opioids continue to be effective in relieving acute and chronic moderate-to-severe pain that occurs after surgical operations [12]. Hence, notwithstanding the scarcity of accessible data, a persistent advantage linked to perioperative oral analgesia exists.

## During surgery phases

### Standard anesthetic protocol

The implementation of a standardized anesthetic protocol is a crucial component of TJR as part of the ERAS protocols, and the specific methods utilized may vary. Spinal anesthesia is widely preferred in clinical settings [4, 13, 14]. It is important to acknowledge conflicting results among the research [13–16]; yet, it is recommended that neuraxial anesthesia be prioritized as the initial choice for patients undergoing knee or hip arthroplasties.

### Local anesthetics for infiltration analgesia and motor sparing nerve blocks

Local infiltration analgesia (LIA) is the most essential pain treatment remedy across the perioperative period. It is worth noting that nerve blocks are not universally regarded as a fundamental element of ERAS protocols [4]. LIA exhibits a notable advantage over nerve blocks due to its absence of motor inhibition, hence promoting earlier ambulation [4]. Nevertheless, it is crucial to consider the potential effects of nerve blocks on motor function and to tailor their use according to the specific physical condition and comorbidities of each patient. The PROSPECT guidelines of the European Society of Regional Anaesthesia & Pain Therapy (ESRA) in 2019 and 2020 provided suggestions pertaining to pain management measures following hip and knee replacement surgery, correspondingly. The fascia iliaca (FI) and adductor canal nerve blocks are indicated as the optimum peripheral nerve blocks for mitigating postoperative pain in patients who are undergoing total hip and total knee replacement surgery, respectively [17]. Nevertheless, there has been a notable increase in the application of advanced ultrasound-guided nerve blocks in the field of clinical practice [18, 19]. Currently, there are sophisticated motor-sparing peripheral nerve blocks that have shown significant effectiveness in preserving quadriceps motor function. These include the pericapsular nerve block (PENG) [20], supra-inguinal fascia iliaca nerve block (s-FIC) [21] and sub-sartorial nerve blocks, such as the adductor canal nerve block. As a result, these treatments have demonstrated benefits for patients in relation to surgical recovery in comparison to traditional methods such as lumbar plexus, femoral, and FI nerve blocks.

### Prevention of perioperative blood loss

As per the established standards, the administration of tranexamic acid (TXA) is recommended to mitigate perioperative blood loss and reduce the requirement for postoperative allogenic blood transfusion [4]. There is a notable association between total knee and total hip arthroplasties and substantial blood loss. These concerns encompass higher rates of surgical infection, delayed physical recovery, extended hospitalization periods, and elevated morbidity and mortality rates [22]. The specific dosage of TXA remains uncertain [23–28], although there is a significant body of evidence supporting its use in TJA, suggesting that it is a recommended approach.

### Maintaining normothermia

It is imperative to employ pre-warming procedures and actively warm patients during both the pre-operative and post-operative phases in order to sustain a normal body temperature [4]. The occurrence of perioperative hypothermia is widely acknowledged as a notable risk factor for the development of postoperative problems, with a special emphasis on its impact on the geriatric population. This syndrome possesses the capacity to elicit irregularities in coagulation and platelet function, raise rates of cardiac morbidity, heighten the probability of surgical site infections and contribute to the manifestation of pressure ulcers [29–37]. Intraoperative utilization of many techniques has been observed, including air warming systems, air-free warming apparatus, reflecting blankets and warmed infusion of fluids. The administration of these medications has been found to reduce the duration of spontaneous breathing, the time it takes for the eyes to open, the recovery of consciousness, and the process of extubation. Additionally, their use has been shown to decrease the occurrence of shivering and postoperative cognitive dysfunction following surgery [29–37]. Despite the paucity of empirical evidence, it is beneficial for patients undergoing TJR to uphold normothermia using diverse modalities throughout the surgical intervention.

### Antimicrobial prophylaxis and skin preparation

The guidelines recommend the utilization of systemic antibacterial prophylaxis for patients having TJR [4]. Infection after surgical procedures involving hip and knee replacements is a comparatively infrequent though noteworthy consequence, necessitating the essential implementation of antibiotic prophylaxis to effectively reduce the occurrence of such infections. Currently, a dearth of universally recognized guidelines and a consensus about the implementation of antibiotic/antiseptic prophylaxis in TJR exists. The utilization of an antimicrobial prophylaxis regimen is a customary procedure in modern orthopaedic surgery, to address the prevalent bacteria linked to infection. The techniques employed in this study involved the administration of cefazolin, cefuroxime, or vancomycin either as monotherapy or in conjunction with gentamicin [38–40]. Therefore, to reduce the risk of infection after TJR, it is advised that patients undergoing TJR should be administered systemic antimicrobial prophylaxis. Ensuring proper skin preparation is crucial as well.

## Perioperative Surgical Factors

### Surgical approach and technique

Different surgical procedures can influence surgery outcomes, complications and the process of recovery. One option that has been identified is minimally invasive surgery, which is thought to have potential benefits such as reduced stress and pain [4]. Nevertheless, the guidelines did not place significant emphasis or officially approve any particular surgical technique due to the absence of conclusive evidence [4]. Overall, the utilization of a minimally invasive approach including a restricted incision has the potential to yield superior results, provided that it is integrated into an ERAS protocol.

### Drainage

To refrain from the routine utilization of surgical drains in hip and knee replacement procedures is well perceived [4]. The application of suction drains after orthopaedic surgery seems to be a reasonable and effective method for reducing the size of postoperative wound hematomas. Previous studies have indicated that the utilization of drains may not be necessary in individuals undergoing total joint arthroplasty [41, 42].

In relation to urine drainage, a clinical investigation has suggested that catheterization may be unnecessary in cases of total knee arthroplasty without drainage when a combination of spinal epidural anesthesia is employed [43]. Despite the relatively modest body of evidence, it is advisable to reduce the usage of frequently utilized drainages and, once introduced, promptly discontinue their use.

### Perioperative fluid management

The preservation of fluid equilibrium is a crucial component of ERAS protocols, particularly in those following TJR. Within this particular context, a suggestion has been put forth advocating for the implementation of fluid management protocols that adhere to either restricted or balanced approaches, while discouraging the use of liberal protocols [4]. The utilization of goal-directed approaches is advocated in the literature [43–47]. Nevertheless, further work is required to determine the exact impact of these treatments.

## After surgery phases

### Thromboprophylaxis

Immediate patient mobilization following surgery in conjunction with proper tromboprophylaxis is of vital importance [4]. Individuals undergoing orthopedic surgery are at an increased susceptibility to develop venous thromboembolism (VTE), a medical disorder that substantially adds to perioperative morbidity and mortality [48]. The combination of pharmacological and mechanical prophylaxis is recommended for mitigating the risk of venous thromboembolism in patients undergoing TJR.

### Nausea and vomiting

The administration of multimodal prophylaxis for postoperative nausea and vomiting (PONV) in patients having hip and knee replacement surgeries involves the use of dexamethasone and/or 5-hydroxytryptamine receptor 3 (5-HT3) antagonists [4]. PONV is a commonly encountered complication that often arises after arthroplasty treatments. This adverse event can have a substantial impact on the patient’s discharge process, as well as worsen their discomfort and emotional distress. The risk factors associated with this illness, as indicated in prior research [49, 50], include bilateral total joint arthroplasty, motion sickness, a history of migraines, a lower body mass index, female gender, non-smoking status and the use of postoperative opioid drugs. Notwithstanding the scarcity of accessible evidence, it is advisable to implement diverse strategies to avoid PONV.

### Nutritional care and intervention

The occurrence of malnutrition substantially augments the probability of in-hospital mortality, postoperative complications, overall mortality and reoperation rates among individuals receiving total hip arthroplasty. Hence, it is imperative to administer thorough nutritional therapy to these individuals to mitigate the elevated postoperative risks [51, 52]. Indeed, the use of suitable nutritional therapies subsequent to the surgical procedure has the potential to yield favorable results.

## Conclusion

The adoption of ERAS protocols has initiated a paradigm shift in orthopedic surgery. A comprehensive investigation has been undertaken regarding ERAS protocols for TJR. Grounded on evidence and data, ERAS protocols have underscored their clinical value in the safety, efficacy and broad applicability in the perioperative period. Orthopedic surgeons have successfully achieved significant reductions in hospital stays, improved patient outcomes and decreased complications by challenging standard methods and accelerating patient care. Future research endeavors must prioritize the examination of the enduring consequences associated with the implementation of standardized ERAS protocols. By doing so, these studies will contribute to the continuous development of orthopaedic surgery and further enhance all outcomes that emphasize the well-being and satisfaction of patients.
